# Evaluation of the Comprehensive Geriatric Assessment (CGA) tool as a predictor of postoperative complications following major oncological abdominal surgery in geriatric patients

**DOI:** 10.1371/journal.pone.0264790

**Published:** 2022-03-03

**Authors:** Yoon Penning, Antoine El Asmar, Michel Moreau, Julie Raspé, Lissandra Dal Lago, Thierry Pepersack, Vincent Donckier, Gabriel Liberale

**Affiliations:** 1 Department of Surgical Oncology, Institut Jules Bordet, Université Libre de Bruxelles (ULB), Brussels, Belgium; 2 Data Centre and Statistics Department, Institut Jules Bordet, Université Libre de Bruxelles (ULB), Brussels, Belgium; 3 Department of Medicine, Institut Jules Bordet, Université Libre de Bruxelles (ULB), Brussels, Belgium; University of Sao Paulo: Universidade de Sao Paulo, BRAZIL

## Abstract

**Introduction:**

The concept of frailty extends beyond chronological age. Identifying frailty using a two-step approach, starting with the use of a screening tool (G8) followed by comprehensive geriatric assessment (CGA), may be useful in guiding treatment decisions and follow-up. This study evaluated the association between G8 and CGA, and the risk of 90-day postoperative complications risk, in oncogeriatric patients.

**Methods:**

Data on geriatric patients with major oncological abdominal surgery was retrospectively collected from our hospital records between 2016 and 2019. Patients with an impaired G8 screening score, who subsequently underwent CGA geriatric screening, were included. Postoperative complications were classified using the Clavien-Dindo classification (CD), and the Comprehensive Complication Index (CCI). The association between the individual components of the geriatric assessment tools and the 90-day postoperative complications risk was analyzed.

**Results:**

One hundred and twelve patients, aged ≥ 70 years, operated for an intra-abdominal tumor with curative intent, were included. Seventy-six patients (67.9%) presented with an impaired G8, out of whom sixty-six (58.9%) had a CGA performed. On univariate analysis, altered nutritional status assessed by the Mini-Nutritional Assessment-Short Form was the only variable associated with higher postoperative total complication rate (*p = 0*.*01*). Patients with an impaired G8 had significantly more postoperative complications and higher 1-year mortality rates than patients with normal G8. Fifteen patients (13.4%) had grade III-IVb complications. A CCI > 50 was recorded in 16 patients (14.3%). All-cause 90-day postoperative mortality was 10.7%.

**Conclusion:**

Identifying an altered preoperative nutritional status, as part of the CGA, in patients screening positive for frailty, is a potentially modifiable risk factor that can enhance preoperative management and optimize treatment decision making. G8 may be a predictive factor for postoperative complications in oncogeriatric patients.

## Introduction

The age distribution of a population greatly affects its burden of disease and disability, including cancer incidence, morbidity, and mortality [1]. With the sharp increase in life expectancy observed in both men and women, virtually every country in the world is experiencing growth in the size and proportion of older people in their population. Over the next three decades, the global number of older people is expected to more than double, reaching over 1.5 billion by 2050, with up to 16% of the world’s population being 65 years and above [2].

Although the relationship between ageing and cancer is complex and far from understood, the incidence of cancer increases with age, as seen in humans and in animal experimental models [3]. As global epidemiologic and demographic transitions continue, they signal an ever-growing cancer burden over the next few decades, with over 20 million new cases expected annually as of the year 2025. With surgical intervention being the main curative treatment for many solid tumors, the number of older patients undergoing surgery as part of their cancer therapy regimen is also expected to rise [4].

These older patients are often considered at increased risk for complications after major surgery but chronological age alone is not a reliable predictor of postoperative complications, as it cannot on its own, capture the physiologic heterogeneity prevalent in this population [5, 6]. The concept of frailty extends beyond chronological age and is one of the most serious global health challenges to be faced in the coming century. Frailty can be defined as “a complex clinical condition characterized by a decline in physiological capacity and reserve across several organ systems, with a resultant increased susceptibility to stressors” [7]. Older patients considered fit for surgery might do as well as younger patients but frail and vulnerable patients are at an increased risk of adverse postoperative outcomes [8]. The usual method to identify frailty is to evaluate an older patient’s general condition and risk of adverse outcomes using the evidence-based process of comprehensive geriatric assessment (CGA). Through a battery of standardized and validated assessment instruments, CGA evaluates nutrition, cognition, functional status, comorbidities, and geriatric syndromes to identify at-risk patients and to possibly guide management, treatment, and follow-up [9].

However, performing a full CGA is time-consuming, therefore the International Society of Geriatric Oncology (SIOG) recommends a “two-step approach”. This strategy starts with the use of a screening tool to identify patients in need of further evaluation by CGA. Screening can be done with the Geriatric 8 (G8), a screening tool that includes seven items from the Mini Nutritional Assessment (MNA) scoring system, and an age-related component (<80, 80–85, or >85 years). Final scores range from 0 to 17, with a score below 14 indicating a geriatric risk profile [10]. Some components of the CGA have been found to be consistently associated with adverse postoperative outcomes; however, based on current evidence, it is not possible to reach a consensus as to how an optimal geriatric assessment (GA) should be conducted and recommendations vary based on patient age, type of surgery (i.e., minor versus major), and other risk factors [8, 11]. Moreover, geriatric screening using the G8 assessment tool has been reported to be a powerful outcome predictor in surgical oncogeriatric patients in terms of hospital stay, rate of postoperative delirium, and 1-year mortality rates [12].

The purpose of this study was to identify independent predictors of postoperative complications in oncogeriatric patients, based on components of the comprehensive geriatric assessment.

## Patients and methods

### Study design & objectives

This is a monocentric retrospective study, including patients ≥70 year-old operated with curative intent, for an abdominal malignancy, at Institut Jules Bordet (IJB), between January 2016 and December 2019. The study protocol was reviewed and approved by the Ethics Committee of IJB (CE3277). All patients’ data was retrieved from the Institute’s electronic medical records software (Oribase).

The primary objective of the study was to identify whether some components of the CGA are independent predictors of postoperative complications in oncogeriatric patients.

The secondary objective was to evaluate the predictive value of G8 in postoperative complications, between patients identified as frail due to an impaired G8 score and those with a normal G8 score.

### Surgical interventions and patients’ characteristics

Major surgical interventions were defined based on the extent of dissection: major body cavity opened (e.g. peritoneal cavity) and as oncological surgeries with major-to-severe tissue trauma (e.g. resection of an organ or part of it and/or digestive anastomosis). Inclusion criteria comprised patients 70 years or older, scheduled for major (open or laparoscopic) oncological abdominal surgery, such as: hepatectomy, colectomy, abdomino-perineal resection, cytoreductive surgery with or without hyperthermic intraperitoneal chemotherapy (HIPEC), low anterior resection, small bowel resection, exploratory laparotomy, esophagectomy, and gastrectomy. Further inclusion criteria among those patients were: pre-operative geriatric 8 (G8) screening, and in patients scoring below or equal to 14 when evaluated by the G8 screening tool, a comprehensive geriatric assessment score (CGA), performed at least 3 months prior to surgical intervention. All other patients were excluded (e.g. emergency surgeries).

The institutional electronic medical record system (Oribase) was used as the data source. Among different components of the CGA, we retained a set of seven validated scores and questionnaires:

- Katz’s activities of daily living (ADL) and Lawton’s instrumental activities of daily living (IADL) scales to assess functional status [13, 14]
- Mini-Mental State Examination (MMSE) to assess cognitive status [15]
- Geriatric Depression Scale (GDS) to assess depression status [16]
- Mini-Nutritional Assessment Short Form (MNA-SF) to assess nutrition status [17]
- Hospital Anxiety and Depression Scale (HADS-A HADS-D) to assess the symptom severity of anxiety disorders and depression [18].
- Patients’ clinical and demographic characteristics were retrieved as well: age, gender, cancer type and histology, type of intervention, G8/ CGA results, and complications.

### Outcomes

Postoperative complications obtained from medical records were defined as any event requiring treatment occurring in a 90-day period after the intervention. Severity of complications were classified by the primary investigator according to the Clavien-Dindo (CD) classification scale (grades I to V) [19]. Grades I and II were considered minor complications whereas grades IIIa to IVb were recorded as major complications. Grade V meant death of the patient. For complications requiring more than one treatment method, the highest severity grade was noted. In case of multiple complications in a patient, each was recorded and graded separately and, additionally, the Comprehensive Complication Index (CCI) was calculated and reported on an interval scale from 0 to 100 to summarize all (minor and major) complications [20]. Postoperative mortality was defined as death within 90 days.

### Geriatric assessment

In the subgroup of patients scoring below or equal to 14 at G8 survey and having received a CGA, patients were divided into two groups based on the median values of continuous variables for each score and questionnaire. Dichotomized outcome variables for 90-day postoperative complications were created according to CD classification: no complications versus any complications. The impact of the collected variable (assessment tool of the CGA) upon the variable of dependent interest (postoperative complications) was explored by univariate analysis and by fitting logistic regression models using the six subscales of the geriatric assessment.

### G8 screening tool

Development of complications was compared between patients with a G8 ≤14 and a G8 >14. This study investigated postoperative outcomes using binary measures: 1. Any morbidity (grade I-V) within 90 days of surgery, 2. Major morbidity (CD grade ≥ IIIa) within 90 days of surgery, 3. Major CCI score (CCI > 50) 4. Death (CD grade V) within 90 days of surgery.

### Statistical analysis

A descriptive analysis of clinical and demographic variables was performed. To test the significance of each variable in relation to the outcomes, univariate analyses were performed using the chi-square test or Fisher’s exact test. Statistical information was encoded anonymously into a database using Microsoft Excel spreadsheets. A value of *p <0*.*05* was considered to be statistically significant. All analyses were done using the Statistical Package for the Social Sciences (SPSS, version 27.0, New York, US).

## Results

### Baseline patient characteristics

**Fig 1:**
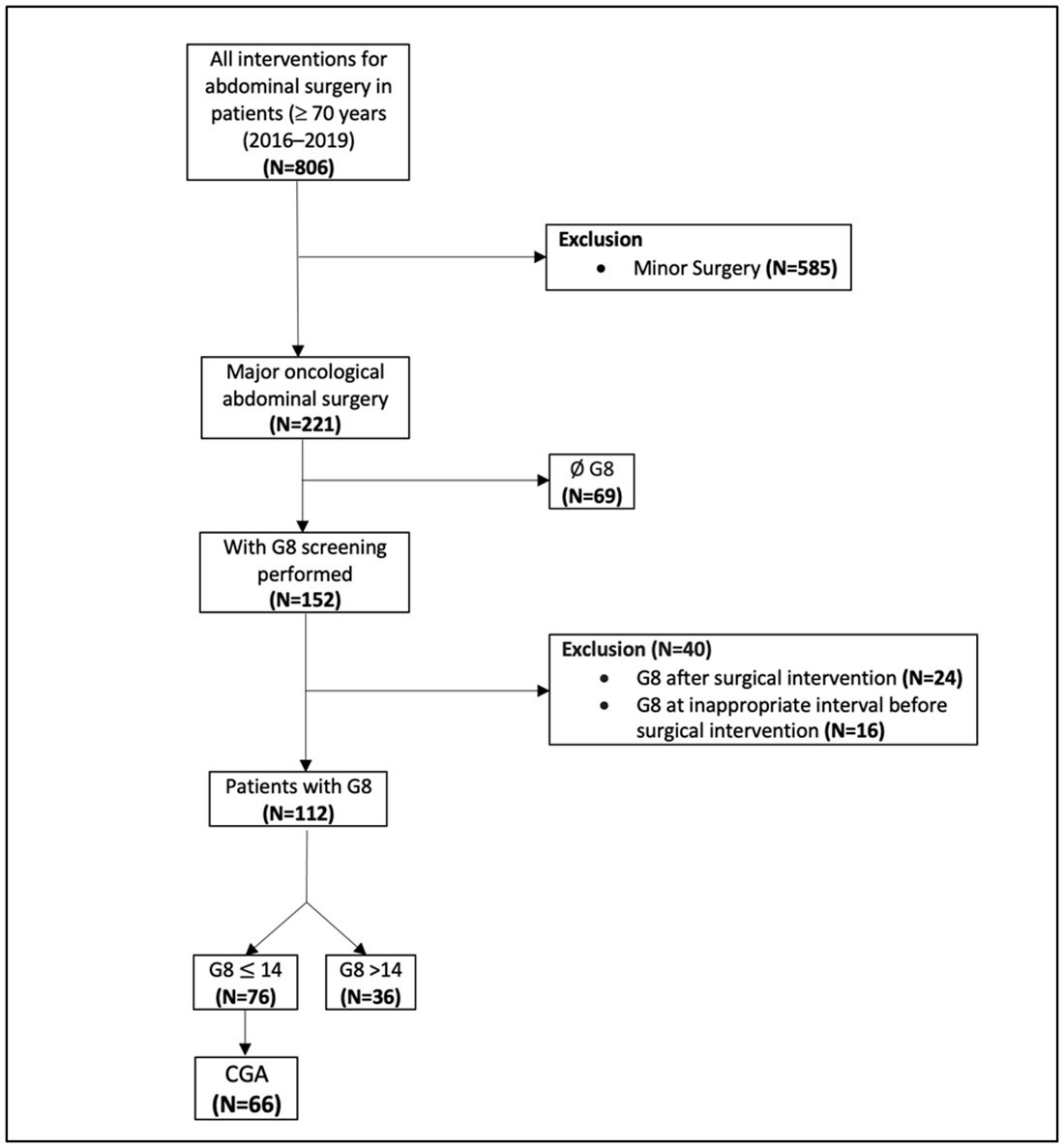
Patients’ flow chart and inclusion criteria.

A total of 1377 patients underwent an elective surgery at our institute during the study period. 806 were ≥ 70 years old, out of whom 112 patients matched the inclusion criteria (Fig 1). Non-oncological surgeries (e.g. cholecystectomy, umbilical or inguinal hernias) and oncological surgeries with a minimal or low amount of tissue trauma and limited extent of dissection (e.g. digestive stoma), were excluded (1156). Furthermore, patients younger than 70 years (571), at the time of surgery, were excluded as well. From the 221 interventions for oncological abdominal surgery remaining, 69 were further excluded because no G8 screening was performed. Of the remaining 152, 40 were excluded for not having an appropriate G8 (performed more than 3 months prior to the intervention, or, after the intervention).

Amongst the 112 patients included, the median age was 74 years, 46 were males (41.1%) and 68 females (58.9%). The most common tumors were colorectal (52.7%) and ovarian (13.4%), and the most common histological type was adenocarcinoma in 84.8% of cases. The majority of surgeries (71.4%) were open surgeries and the most frequent types of interventions were colectomy (25.9%) and cytoreductive/ debulking surgeries (17.9%) ± HIPEC. Malignancies classified as “other” included unknown primaries, endometrial tumors, pancreatobiliary cancers, retroperitoneal cancers and breast, lung, and skin cancers metastatic to the abdomen. The majority of malignancies (37.5%) were grade II. Details of the baseline clinical variables are summarized in Table 1. G8 was impaired in 76 patients (67.9%) and normal in 36 patients (32.1%). When we compared the characteristics of the two groups according to sex, cancer type, tumor histology, type of intervention, and surgical approach, we did not find significant differences except for age and cancer grade (*p = 0*.*04*).

**Table 1:** Patients’ clinical and demographic characteristics, and their correlation with an impaired G8 with respect to each subgroup.

Variable	Total N(%)	G8 < = 14 n (%)	G8 > 14 n (%)	Correlation between patients’ characteristics and an Impaired G8
				*p* value
Age	74 (70–92)*	77.4 (5.6)**	75.0 (4.4)**	***0*.*03*** ^†^
Gender				
Female	66 (58.9)	46 (69.7)	20 (30.3)	*0*.*62*
Male	46 (41.1)	30 (65.2)	16 (34.8)	
Cancer Type				
Colorectal	59 (52.7)	39 (66.1)	20 (33.9)	*0*.*88*
Ovary	15 (13.4)	11 (73.3)	4 (26.7)	
Liver	6 (5.3)	4 (66.7)	2 (23.3)	
Stomach	9 (8.0)	5 (55.6)	4 (44.4)	
Small Bowel	2 (1.8)	2 (100)	0	
Esophagus	5 (4.5)	3 (60)	2 (40)	
Other	16 (14.3)	12 (75)	4 (25)	
Histology				
Adenocarcinoma	95 (84.8)	63 (66.3)	32 (33.7)	*0*.*59*
Hepatocellular carcinoma	5 (4.7)	3 (60)	2 (40)	
Soft tissue sarcoma	2 (1.8)	2 (100)	0	
Cholangiocarcinoma	2 (2.8)	2 (100)	0	
Epidermoid Carcinoma	3 (2.7)	3 (100)	2 (40)	
Other	5 (4.5)	3 (60)		
Type of intervention				
Colectomy (open or laparoscopic)	29 (25.9)	19 (65.5)	10 (34.5)	*0*.*53*
Cytoreductive Surgery (without HIPEC***)	20 (17.9)	12 (60)	8 (40)	
Hepatectomy (open or laparoscopic)	16 (14.3)	11 (68.8)	5 (31.2)	
Low Anterior Resection (rectum)	12 (10.7)	7 (58.3)	2 (20)	
Exploratory Laparotomy	10 (8.9)	8 (80)	0	
Cytoreductive Surgery (with HIPEC)	5 (4.5)	5 (100)	3 (37.5)	
Abdominoperineal Resection	8 (7.1)	5 (62.5)	3 (50)	
Gastrectomy (any)	6 (5.3)	3 (50)	0	
Esophagectomy (total or subtotal)	3 (2.7)	3 (100)	0	
Small Bowel Resection	3 (2.7)	3 (100)		
Cancer grade				
I -	29 (25.9)	18 (62.1)	11 (37.9)	***0*.*04***
II	42 (37.5)	29 (69.1)	13 (30.9)	
III	28 (25.0)	23 (82.1)	5 (17.9)	
Unknown	13 (11.5)	6 (46.2)	7 (53.8)	
Type of surgery				
Open	80 (71.4)	57 (71.3)	23 (28.7)	*0*.*2*
Laparoscopic	32 (28.6)	19 (59.4)	13 (40.6)	

^**†**^Student T-test;

*Median age (range);

**Mean age (standard deviation);

***HIPEC: Hyperthermic intra-peritoneal chemotherapy.

### Comprehensive geriatric assessment—CGA

Among the 76 patients with an impaired G8, the median age was 76 years (70–92 years). Ten of these patients were not evaluated by CGA at all. Preoperative CGA scores, among the remaining 66 patients, were ADL > 6 in 33.3%, IADL ≤ 7 in 50.8%, MMSE ≤ 28 in 57.9%, GDS > 3 in 50.0%, HADS-A > 6 in 42.9%, HADS-D > 4 in 46.4%, and MNA-SF ≤ 9 in 39.4%.

### Postoperative outcomes

Out of all 112 patients, 77 developed at least one complication. Forty-three patients (38.4%) experienced a grade II complication and 15 patients (13.4%) developed a major complication. Sixteen patients (14.3%) had a CCI score > 50. Twelve patients (10.7%) died within the 90-day follow-up period from all causes and from postoperative complications. Postoperative Clavien-Dindo scores are displayed in Table 2.

**Table 2:** Postoperative Clavien-Dindo scores in oncogeriatric patients, according to their G8 score.

Patients	0	I	II	IIIa	IIIb	IVa	IVb	V
**G8 ≤ 14 (N = 76)**	18	5	33	6	3	1	0	10
**G8 > 14 (N = 36)**	17	2	10	3	2	0	0	2
**Total**	35	7	43	9	5	1	0	12

### Association between CGA and complications

We analyzed the impact of each component of the CGA upon the development of complications. In univariate analysis, the short form of the Mini-Nutritional Assessment was the sole prognostic factor for postoperative complications (*p = 0*.*03*). There were no associations between the other assessment tools and the emergence of complications. Results are shown in Table 3.

**Table 3:** Univariate analysis for each CGA component score, as predictors of postoperative complications.

CGA⸰ Component Score	Patients Total N^‡^—%*	Complications		*p* value
		Yes N—%**	OR (95% CI)***	
**ADL**				
< 6	44–66.7%	33–75%	1	
≥ 6	22–33.3%	19–86.4%	2.1 (0.5–8.5)	*0*.*35*
**IADL**				
< 7	33–50.8%	25–75.8%	1	
≥ 7	32–49.2%	26–81.3%	0.7 (0.2–2.4)	*0*.*59*
**MMSE**				
< 28	34–59.6%	25–73.5%	1	
≥ 28	23–40.4%	20–87.0%	2.5 (0.6–10)	*0*.*32*
**GDS**				
< 3	41–68.2%	33–80.5%	1	*1*
≥ 3	20–32.8%	16–80.0%	0.97 (0.3–3.3)	
**HADS-A**				
< 6	33–58.9%	26–78.8%	1	
≥ 6	23–41.1%	18–78.3%	0.97 (0.3–3.3)	*1*
**HADS-D**				
< 4	30–53.6%	22–84.6%	1	
> 4	26–46.4%	22–73.3%	0.5 (0.1–1.9)	*0*.*30*
**MNA-SF**				
< 9	26–39.4%	28–70.0%	1	
≥ 9	40–60.6%	24–92.3%	5 (1.04–25)	***0*.*03***

**⸰**CGA: comprehensive geriatric assessment;

^**‡**^N: total number of patients;

* Relative percentage to the total number of patients;

** Relative percentage within each category;

***OR: Odds ratio; CI: Confidence interval.

### Association between G8 and complications

Table 4 provides an overview of the univariate analysis for predictors of complications, higher CD-score, higher CCI score, and mortality. A G8 score below or equal to 14 was significantly associated with the development of complications within 90 days of surgery. Patients who screened positive for frailty had significantly more complications than patients who screened negative, 52% versus 17% respectively (*p<0*.*05*). However, impaired G8 was not associated with the occurrence of major complications or higher CCI score. Additionally, no association was reported between impaired G8 and 90-day mortality, but it was significantly associated with a higher 1-year mortality (*p = 0*.*01*).

**Table 4:** Univariate analysis for predictors of complications, higher CD-Score, higher CCI score, 90-Day and 1-year mortality.

Variables	Complications			Minor vs Major Complications			CCI** score			90-day mortality (Grade V)			1-year mortality		
	Yes	No	*p-*value	≤ II	III-IVb	*p-*value	≤ 50	> 50	*p-*value	Yes	No	*p-*value	Yes	No	*p-*value
Screening															
G8 ≤ 14 (76)*	58	18	**0.01**	56	10	0.95	63	13	0.21	10	60	0.18	23	53	**0.01**
G8 > 14 (36)*	19	17		29	5		33	3		2	34		3	33	

*Total number of patients within each category;

**CCI: Comprehensive Complication Index.

## Discussion

The primary aim of our study was to identify independent predictors of postoperative complications based on components of the CGA. In our population, 67.9% of the patients were identified by an impaired G8 as being at risk for frailty. Among those patients, 26 scored below or equal to 9 when evaluated by the short form of the Mini-Nutritional Assessment tools. Our main finding was the strong association between this nutrition-based evaluation tool and the occurrence of any complications during the 90-day postoperative course. In this group of patients with an abnormal G8 score, no association was found between other components of the CGA and postoperative complications. This relationship has been increasingly investigated in the field of surgical oncology but with inconsistent results. In patients with colorectal cancer, ADL, IADL, MMSE, GDS, and MNA were inconsistently associated with postoperative complications [9]. Huisman et al. found “impaired nutrition” to be associated with major postoperative complications, but the authors evaluated the nutritional status using the Nutritional Risk Screening (NRS) in their study [8]. For patients undergoing gastric cancer resection, other malnutrition screening tools have been validated and found to be associated with peri-operative and postoperative morbidity and complications [21].

Our findings indicate the importance of preoperative nutritional assessment in major oncological abdominal surgery. This tool provides valuable patient information, to assess the surgical risk-benefit ratio, and to possibly tailor an individualized nutritional therapy by a multidisciplinary team, even though the impact of normalization of potentially reversible factors on postoperative complications is still under investigation [22]. However, despite recommendations on geriatric assessment in the oncogeriatric patients’ population, published in 2005 by the SIOG, there is still no consensus regarding which specific instruments should be included in a CGA [12]. Inclusion of nutritional impairment screening is of high interest as this reversible factor seems to be an indicator of increased postoperative morbidity. Heterogeneity between patient selection and surgical intervention makes comparison difficult and indicates the need for further studies, with larger populations, using identical screening tools and cut-off values.

Another important finding in our study was the significant difference in postoperative outcomes between patients with a G8 ≤ 14 and patients with a G8 > 14. We identified an association between impaired G8 screening and the development of at least one complication. Several studies investigating this relationship have provided contradictory results. A recent study on preoperative frailty assessment in 114 patients found the G8 tool not to be significantly associated with the risk of adverse events [23]. Another study found similar results in 139 older patients treated surgically for colorectal cancer. The authors did not find isolated G8 to be of any predictive value on postoperative outcomes. They did, however, find that a combination of the G8 and the Identification of Seniors at Risk-Hospitalized Patients (ISAR-HP) screening tool resulted in a high predictive value for postoperative complications [24]. Another combination recently investigated by Bessems et al. indicated that frailty screening by G8 in association with the 4-meter gait speed test predicts postoperative complications in colorectal cancer patients undergoing elective surgery [25]. Additionally, in another study, de Vries et al. found G8 to be a strong predictor of postoperative complications in a population of patients undergoing surgery for cutaneous head and neck cancer [26].

Our study did not find a relation between impaired G8 and major complications (defined as Clavien-Dindo grade > II). When investigating this same relationship in 143 patients of the same age group, requiring surgery for a suspected solid malignancy, Bruijnen et al., found no difference in the occurrence of major 30-day complications [27]. However, three studies did describe an association between G8 and the occurrence of major Clavien-Dindo complications. Studies including grade II complications found this association in 78 patients who underwent surgery for colorectal cancer [9], 184 patients who underwent emergency abdominal surgery (including non-oncological patients) [28] and 71 patients treated for hepatocellular carcinoma [12], thus confirming these findings. In these studies, no association was found between impaired G8 and a higher 1-year mortality rate.

We found impaired G8 to not be predictive of 90-day mortality in our population. It was, however, associated with an increased 1-year mortality. This may be explained by the significantly higher cancer grades in this group.

The predictive value of the G8 on postoperative complications in surgical oncology and practice remains unclear and further trials on larger populations are needed to complete our understanding of this screening tool and its place in diagnostic algorithms. This is in contrast to studies done in non-surgical oncological patients, in whom impaired G8 has been shown to provide helpful information through prediction of complications in patients receiving chemotherapy and/or radiotherapy [29]. Furthermore, it is imperative to mention that the G8 was initially developed as a frailty screening tool for predicting deficits in the CGA. It was not intended to be used as a prognostic tool.

Direct comparison between these study results and ours should be approached with caution. The above-mentioned studies focused mostly on homogenous populations with a single malignancy or surgical intervention whereas our study included a variety of tumor types and surgeries. Heterogeneity remains among patient characteristics between studies, definitions of frailty, cut-off values for screening and assessment tools but also in the definition of adverse postoperative outcomes.

The present study had several limitations. First, although the inclusion criteria limited our population to patients undergoing ‘major’ oncological abdominal surgery, heterogeneity affecting risk of complications remained. For example, Kothari et al. recently showed that within each of their frailty cohorts, laparoscopic colectomy provided better outcomes when compared to an open approach in all domains, including cardiac/vascular, pulmonary, and wound complications [30]. Additionally, this study did not account for differences in tumor characteristics, such as stage, treatment course, or intensity. Another limitation is the monocentric nature of our study since variations in postoperative complications are influenced by technical skill scores of surgeons and quality of care by medical staff [31, 32]. Other limitations lie in the fact that postoperative outcomes were collected retrospectively. As a result, some minor complications may have been underreported. Despite these limitations, our study was unique in including only geriatric patients admitted for major oncological abdominal surgery, while not being centered around a single malignancy or surgical approach.

## Conclusion

To conclude, this study suggests that the MNA-SF is a valuable asset in preoperative risk assessment for postoperative complications that have the potential to impede recovery in oncogeriatric patients undergoing major abdominal surgery. Evaluation of nutritional status as part of the comprehensive geriatric assessment in patients identified as frail seems essential as nutrition is a potentially modifiable factor influencing preoperative management and treatment modalities. G8, besides its role as a screening tool for impairment in the CGA, shows potential as a predictor of postoperative complications. This finding corroborates well with the MNA’s predictive potential since all the questions of the G8 –except for age–are derived from the MNA.
